# Implementation of a Cardiogenic Shock Team and Clinical Outcomes (INOVA-SHOCK Registry): Observational and Retrospective Study

**DOI:** 10.2196/resprot.9761

**Published:** 2018-06-28

**Authors:** Behnam Tehrani, Alexander Truesdell, Ramesh Singh, Charles Murphy, Patricia Saulino

**Affiliations:** ^1^ Inova Heart and Vascular Institute Falls Church, VA United States; ^2^ Virginia Heart Falls Church, VA United States

**Keywords:** cardiogenic shock, mechanical circulatory support

## Abstract

**Background:**

The development and implementation of a Cardiogenic Shock initiative focused on increased disease awareness, early multidisciplinary team activation, rapid initiation of mechanical circulatory support, and hemodynamic-guided management and improvement of outcomes in cardiogenic shock.

**Objective:**

The objectives of this study are (1) to collect retrospective clinical outcomes for acute decompensated heart failure cardiogenic shock and acute myocardial infarction cardiogenic shock, and compare current versus historical survival rates and clinical outcomes; (2) to evaluate Inova Heart and Vascular Institute site specific outcomes before and after initiation of the Cardiogenic Shock team on January 1, 2017; (3) to compare outcomes related to early implementation of mechanical circulatory support and hemodynamic-guided management versus historical controls; (4) to assess survival to discharge rate in patients receiving intervention from the designated shock team and (5) create a clinical archive of Cardiogenic Shock patient characteristics for future analysis and the support of translational research studies.

**Methods:**

This is an observational, retrospective, single center study. Retrospective and prospective data will be collected in patients treated at the Inova Heart and Vascular Institute with documented cardiogenic shock as a result of acute decompensated heart failure or acute myocardial infarction. This registry will include data from patients prior to and after the initiation of the multidisciplinary Cardiogenic Shock team on January 1, 2017. Clinical outcomes associated with early multidisciplinary team intervention will be analyzed. In the study group, all patients evaluated for documented cardiogenic shock (acute decompensated heart failure cardiogenic shock, acute myocardial infarction cardiogenic shock) treated at the Inova Heart and Vascular Institute by the Cardiogenic Shock team will be included. An additional historical Inova Heart and Vascular Institute control group will be analyzed as a comparator. Means with standard deviations will be reported for outcomes. For categorical variables, frequencies and percentages will be presented. For continuous variables, the number of subjects, mean, standard deviation, minimum, 25th percentile, median, 75th percentile and maximum will be reported. Reported differences will include standard errors and 95% CI.

**Results:**

Preliminary data analysis for the year 2017 has been completed. Compared to a baseline 2016 survival rate of 47.0%, from 2017 to 2018, CS survival rates were increased to 57.9% (58/110) and 81.3% (81/140), respectively (*P*=.01 for both). Study data will continue to be collected until December 31, 2018.

**Conclusions:**

The preliminary results of this study demonstrate that the INOVA SHOCK team approach to the treatment of Cardiogenic Shock with early team activation, rapid initiation of mechanical circulatory support, hemodynamic-guided management, and strict protocol adherence is associated with superior clinical outcomes: survival to discharge and overall survival when compared to 2015 and 2016 outcomes prior to Shock team initiation. What may limit the generalization of these results of this study to other populations are site specific; expertise of the team, strict algorithm adherence based on the INOVA SHOCK protocol, and staff commitment to timely team activation. Retrospective clinical outcomes (acute decompensated heart failure cardiogenic shock, acute myocardial infarction cardiogenic shock) demonstrated an increase in current survival rates when compared to pre-Cardiogenic Shock team initiation, rapid team activation and diagnosis and timely utilization of mechanical circulatory support.

**Trial Registration:**

ClinicalTrials.gov NCT03378739; https://clinicaltrials.gov/ct2/show/NCT03378739 (Archived by WebCite at http://www.webcitation.org/701vstDGd)

## Introduction

### Background and Significance

Cardiogenic shock (CS) is the strongest predictor of mortality in patients who experience an acute myocardial infarction (AMI) or who suffer an episode of acute decompensated heart failure (ADHF). Observational studies have indicated that patient populations particularly at risk of developing cardiogenic shock post AMI or post an episode of ADHF include the elderly and patients with concurrent cardiovascular comorbidities [1]. Advances in efforts to provide early identification of CS, revascularization, and restored perfusion have positively affected mortality rates associated with myocardial infarction (MI) and have caused a dramatic fall in deaths associated with AMI in recent decades, while in the patients who develop CS post MI, mortality continues to be persistent and remains as high as 50% [2].

CS, also known as “pump failure” is precipitated by a profound reduction in cardiac output which results in tissue hypoperfusion secondary to a deficit of circulating blood, this lack of perfusion results in increasingly poor clinical outcomes [2,3,4]. Also defined as complete circulatory collapse, CS is characterized by shock occurring after a primary cardiac pathology in which cardiac output has been compromised. It involves refractory hypotension and tissue hypoperfusion secondary to heart failure after correction of preload and the culprit precipitating arrhythmia [2]. CS is representative of a diverse and complex clinical presentation which is the challenge of identification and management of this calamity. The onset of CS can be acute or progressive, as the ischemia representative of CS may develop from an acute, large, primary MI or can occur as a delayed extension of an original MI [2]. CS can occur acutely in patients with no prior history of cardiac disease, or progressively in patients with chronic heart failure [4]. The most common etiology of acute CS is the incidence of an acute coronary syndrome (ACS), specifically ST-segment elevation myocardial infarction (STEMI) or non-ST-segment elevation myocardial infarction (NSTEMI) which collectively has been reported to account for nearly 80% of cardiogenic shock cases [4]. Patients that develop CS post NSTEMI tend to develop dormant CS and are older with more complex cardiac comorbidities [2]. CS is the strongest predictor of mortality post MI. This is thought to result from both ischemic and mechanical complications. Mechanical complications of MI that contribute to CS include: acute mitral regurgitation secondary to rupture of the papillary muscle, rupture of the ventricular septal wall, or tearing of the ventricular wall [4]. CS can also arise from non-ACS causes such as cardiac abnormality (primary, valvular, electrical, or pericardial), decompensated valvular disease, acute myocarditis, hypertrophic cardiomyopathy with aortic obstruction, cardiac tamponade, arrhythmia, traumatic injury to the chest (myocardium), post cardiotomy secondary to coronary artery bypass graft surgery, and progression of congenital lesions [4].

A designated, multidisciplinary shock team is critical in the assessment, implementation, and management of the CS patient. The collaborative efforts of a team which includes a cardiologist specializing in heart failure, a cardiothoracic surgeon, cardiac inteventionalist, and an intensive care unit (ICU) intensivist working to manage the time-dependent clinical scenario will maximize outcomes related to CS [5]. The Cardiogenic Shock team at the Inova Heart and Vascular Institute (IHVI) is comprised of four clinical disciplines: Interventional Cardiology, Acute Heart Failure, Cardiac Surgery, and Critical Care. Their respective roles in the diagnosis, treatment and management of cardiogenic shock are highly specific yet profoundly collaborative. Interventional cardiology is at the vanguard of treatment and is usually the first point of contact. This group assists with mechanical circulatory assist device insertion and management both on hospital admission and throughout hospitalization. They also manage device weaning and escalation of support. The acute heart failure physician specialist helps to determine a patient’s candidacy for temporary and durable mechanical support. The role of the cardiac surgeon is to assist with and evaluate a patient’s candidacy for surgical support services. Finally, the critical care physician is the team quarterback and the central player in identifying the appropriate parties for emergency consultation as well as the advancement and management of the cardiogenic shock patient’s day-to-day care during hospitalization. IHVI CS team goals can be identified as follows: rapid identification of CS as well as its etiology, maximization of survival through the utilization of mechanical circulatory support (intraortic balloon pump; Impella, percutaneous microaxial flow pump; Tandem Heart, percutaneous left ventricular assist device; peripheral extracorporeal membrane oxygenation [ECMO]; central ECMO; temporary vascular assist device; permanent vascular assist device; or transplant) as well as supportive therapies, and the development and implementation of a hemodynamic support plan with mechanical devices in the event of refractory CS [5]. Team-based interventions are crucial in critical illness as in the case of a “code team” (mandated by the Joint Commission on Accreditation of Healthcare Organizations) for the in-house management of cardiac arrest and a rapid response team for decompensating medical surgical patients. CS is similar to these clinical clusters of symptoms and requires the early identification and specific expertise of many disciplines in order to manage this complex condition. The high mortality rate of CS patients can be tempered through early revascularization and the activation and utilization of a multidisciplinary shock team. The time sensitive nature of CS or the dictum “time to support” [6], with both percutaneous and surgical interventions, requires the activation of a multidisciplinary Shock team in order to manage circulatory collapse and the ensuing end organ dysfunction [6] or failure through prompt response and management of any changes in the patient’s condition in addition to early diagnosis.

A review of relevant literature identifies the benefit and recommends the implementation of a multidisciplinary shock team to improve outcomes in patients in danger of imminent circulatory collapse. Proposed recommendations are wide in scope yet highly specialized with respect to the requirements of this patient population. CS associated with acute MI or acute decompensated heart failure should be closely monitored for progression to decompensation and end organ failure. CS should be suspected and investigated post cardiac arrest due to the significant association between the two conditions by a multidisciplinary shock team [7]. Recommended personnel include a multidisciplinary oversight panel as well as experienced medical teams at a given site [7]. Medical, interventional cardiology, anesthesia, thoracic and vascular surgery, intensive care, and radiology [7] must be available to manage CS in a timely manner. The two strongest priorities in CS are hemodynamic stabilization and the rapid reversal of the low output state to maintain end organ perfusion and rapid coronary reperfusion, although not necessarily in that order, are optimally managed by a CS team [8,9]. Rehospitalization and death are most prevalent in the early discharge period [10]. These readmissions are frequently associated with volume overload as opposed to late readmissions which are associated with the natural trajectory of the syndrome for example cardiac remodeling [10]. This identifies the early discharge period as one where the patient is particularly vulnerable and the careful coordination of the multidisciplinary team as well as the transitional team are implemented to ensure that the care is patient-centered, as well as proactive in protection against recurrence of CS [10]. Activation of a coordinated cardiogenic shock team and early outcome specific therapy in a timely and synchronized manner ensures proper allocation of resources and is associated with increased survival in cardiogenic shock [11]^.^ Successful device selection to support heart rate (HR) in underlying CS etiologies is enhanced by a multidisciplinary team approach including; heart failure specialists, interventional cardiologists, and cardiothoracic surgeons, with patients’ preferences accommodated in a timely manner [12].

The implementation and utilization of a designated CS at the IHVI is an effective strategy to mitigate the consequences of cardiogenic shock. The IHVI CS team, founded in 2017, is mobilized for and directed to five goals: (1) the rapid identification of shock through early activation of the CS team and rapid collaborative decision making, (2) early right heart catheterization to facilitate invasive hemodynamic therapy tailored to the patients’ unique presentation, (3) the accelerated initiation of mechanical circulatory support, (4) minimization of vasopressor and inotropic support, and (5) meaningful recovery and survival of the patient [13]. Bi-weekly action and outcomes review are employed to track efficient methodology with the intention of eradicating significant preventable morbidity and death from CS [13]. Utilizing the best and most recent evidence and best practices, a comprehensive care pathway was developed. A six-month training process was focused on individual and team management of the aforementioned five goals, training and rehearsals were implemented to ensure the seamless execution of the adopted algorithms [13]. Prior to implementation of the Cardiogenic Shock team at the IHVI, 30-day survival was approximately 47%, assuming an alpha level of 0.5, two sided. We expect the implementation of an IHVI Shock algorithm to increase 30-day survival by 15%. Thus, 200 subjects in each group provide at least 80% power to detect a statistically significant increase in 30-day survival from 47%.

We propose to study the outcomes associated with CS patients who have been managed by the CS team at the IHVI in order to ascertain the effects on outcome and survival rate. Positive effects in outcomes and survival will indicate a positive correlation between meaningful survival and management by a multidisciplinary shock team. The database compiled from these outcomes will also serve as a clinical archive of distinctive patient characteristics and outcomes to support future evaluation and translational research. The benefit of CS management to patients is a decrease in disability and mortality post CS episode.

### Specific Aims

The specific aims of this study are as follows: (1) to collect retrospective clinical outcomes related to acute decompensated heart failure cardiogenic shock, acute myocardial infarction cardiogenic shock and compare current versus historical survival rates; (2) to collect Inova Heart and Vascular Institute (IHVI) site specific outcomes before and after initiation of the Cardiogenic Shock team on January 1, 2017; (3) outcomes related to implementation of mechanical circulatory support versus no circulatory intervention and type of intervention (ECMO versus intracorporeal axial-flow [Impella]); and (4) to assess survival at 3 time points.

### Hypothesis

We hypothesize that implementation of a Cardiogenic Shock initiative with early team activation, rapid initiation of mechanical circulatory support, hemodynamic-guided management, and strict protocol adherence will be associated with superior clinical outcomes at three time points: survival to discharge and overall survival, compared to 2015 and 2016 outcomes prior to shock team initiation.

## Methods

### Study Design and Subject Selection

#### Study Type

This is a retrospective and prospective, observational study. The Investigators acknowledge that a possible limitation of measuring and comparing the treatment effect of the team-based approach with historical controls not exposed to team intervention may present selection bias due to the change in treatment paradigm, potential loss of blinding, lack of true point estimates, and increased risk of type I error.

#### Setting or Location

Outpatient or inpatient chart review utilizing EPIC, the electronic medical record utilized by the Inova Health System (HIS) for patients admitted to Inova Fairfax Medical Campus. EPIC review will occur at the Center for Thrombosis Research and Drug Development Center: 3300 Gallows Road, Inova Heart and Vascular Institute 3rd Floor, Fairfax, VA 22102.

#### Duration of Study

Chart review in EPIC will occur over a one-year period.

#### Number of Subjects

Our goal is to include approximately 200 patients before and 200 patients after initiation of Shock team. The study group will consist of a retrospective review of all patients receiving Shock Team intervention after diagnosis with acute decompensated heart failure cardiogenic shock, or acute myocardial infarction cardiogenic shock, from January 1, 2017 until Institutional Review Board (IRB) filing of protocol. A second group will consist of current patients receiving shock team intervention.

#### Study Population

##### Gender of Subjects

There are no gender-based enrollment restrictions. Subjects will include a distribution based on the demographics of the Northern Virginia population.

##### Age of Subjects

Anyone 18 years or older who underwent CS team intervention for acute decompensated heart failure cardiogenic shock, acute myocardial infarction cardiogenic shock will be included.

##### Racial and Ethnic Origin

There are no race-based enrollment restrictions. Subjects will include a distribution based on the demographics of the Northern Virginia population.

##### Vulnerable Populations

No vulnerable populations will be enrolled in this study.

#### Recruitment

Preliminary chart review in EPIC will be performed on all patients in our practice and those who have a previously undergone Cardiogenic Shock team intervention for acute decompensated heart failure cardiogenic shock, or acute myocardial infarction cardiogenic shock. No recruitment process will be necessary to obtain information.

#### Inclusion Criteria

Patients at the IHVI with documented Cardiogenic Shock team intervention for acute decompensated heart failure cardiogenic shock, or acute myocardial infarction cardiogenic shock.

Specific criteria for the diagnosis of cardiogenic shock is defined by hemodynamic parameters—systolic blood pressure <90 mm Hg, cardiac index <1.8 L/min/m^2^ without pharmacologic support (or >2.2 L/min/m^2^ with support), left ventricular end-diastolic pressure >18 mm Hg or right ventricular end-diastolic pressure >10-15 mm Hg or pulmonary capillary wedge pressure >15 mm Hg—and clinical signs and symptoms of hypoperfusion, such as cool extremities, decreased urine output, and altered mental status.

Enrollment criteria are specific to patients treated at the IHVI for documented cardiogenic shock with activation of the IHVI Cardiogenic Shock team. Cardiogenic shock will have preceded acute decompensated heart failure or acute myocardial infarction etiologies. Patients will include those transferred from offsite hospitals as well as inpatients at the IHVI.

#### Exclusion Criteria

Patients under the age of 18 will be excluded.

Patients assessed to be comorbid (eg, life expectancy less than 6 months) were not deemed to be suitable candidates for temporary mechanical support or long-term, durable mechanical circulatory support and were excluded in principle from data abstraction

### Research Database Participation Eligibility

#### Participant Eligibility Criteria

Any patient with cardiogenic shock, whether inpatient or transferred from another facility, treated at the Inova Heart and Vascular Institute. This includes adults with and without decision making capacity over the age of 18 years old.

#### Informed Consent

Informed consent is waived for the purpose of this research database. The procedural risk involved in this protocol meets the definition of minimal risk as set forth in 45 CFR 46.102 (i) “Minimal risk means that the probability and magnitude of harm or discomfort in the research are not greater in and of themselves than those ordinarily encountered in daily life or during the performance of routine physical or psychological examinations or tests.” Participation in this protocol requires evaluation of medical data from patients who have experienced CS that is mined directly from the participant’s medical record.

#### Registry Data

The primary investigator will designate the data points to be included in the database in order to assess the efficiency of procedures and protocols in the treatment of CS at the IHVI as administered by the Shock team, to identify relationships between treatment and negative morbidity and mortality outcomes, and to monitor and improve quality of care delivered.

In addition to the defined data collected, additional participant data may be collected as needed for a specific study. This additional data would be contained in the participant’s medical records. Examples of this additional data that may be requested for a related additional study would be more detailed clinical data at the time of diagnosis or more detailed disease status data while experiencing cardiogenic shock. In no case will the participant be contacted in order to obtain additional data.

#### Collaboration With Other Registries

To facilitate both national and international research efforts in cardiogenic shock patient’s collaborative studies may occur with other institutions outside of Inova. Detailed patient-level data obtained through the cardiogenic shock database can facilitate hypothesis-driven clinical studies. The information in the database will not only be utilized to drive internal investigator-driven projects but also be used to foster collaborative studies with institutions outside of INOVA. For any collaborative studies with outside institutions, a data use agreement will be in place. Dataset exporting will be restricted to the database manager and the primary investigator. Only a limited data set will be provided which includes dates to help identify clinical events. No other protected health information will be such as name, contact information, medical record identification number nor any other patient- specific identifiers will be provided to these collaborative partners. In addition, collaborative studies using the cardiogenic shock database will not be approved unless authorized by the principal investigator alone and the aforementioned data use agreement has been executed.

#### Data Confidentiality

Access to all information in the Cardiogenic Shock Research Database is securely controlled utilizing passwords and logins at multiple levels. Access to the research data base is limited to relevant IRB approved study team members and to employees with specific job responsibilities related to the database.

Registry participants are assigned a unique identification (UID) number when they are enrolled into the cardiogenic shock database. All protected health information (PHI) will be fully de-identified. The UID contains no identifying information. This UID is utilized to track all participant information in the research database. Protected health information is defined as:

1. Name
2. Geographical subdivisions smaller than State
3. Telephone number
4. Fax number
5. Electronic mail addresses
6. Social security numbers
7. Medical record numbers
8. Health plan beneficiary numbers
9. Account numbers
10. City, state, and country

Or any other specific identifying information will be collected at the time the unique identification number is assigned to ensure that the participant has not been previously registered. Identifying data will be stored in a secure database separate from the research database. This protected information will not be included in data sets used for analysis. The unique identification number will have no identifying information within it. This number will be used to track all information about the participant in the research database.

The identity of the database participants will be kept confidential at all times. All research staff at INOVA will maintain up-to-date training in protection of human subjects. This training is received through the Collaborative IRB Training Initiative program. This is a Web-based training program offered through the University of Miami.

### Endpoints or Outcome Measurements

#### Primary Outcomes

The primary outcomes of this study are to create an archive of retrospective clinical outcomes (acute decompensated heart failure cardiogenic shock, or acute myocardial infarction cardiogenic shock) and compare current versus historical survival rates and to evaluate all-cause mortality at hospital discharge.

#### Secondary Outcomes

Analysis of mortality by subgroup (age, sex, initial etiology, time to presentation, time to treatment, use of mechanical circulatory support, Incidence of major adverse cardiac and cerebrovascular events, and preservation of left ventricular function.

### Statistical Considerations and Data Analysis

#### Sample Size

We are estimating 300-400 patients will be included in this analysis. This size is based on prior smaller studies.

#### Method of Data Analysis

Data will be collected from a chart review and recorded in a database spreadsheet. Statistical analysis will be performed to calculate data collected in this study. Results will be reported using summary tables and will be displayed for each treatment arm. For categorical variables, frequencies and percentages will be presented. For continuous variables, the number of subjects, mean, standard deviation, minimum, 25th percentile, median, 75th percentile and maximum will be presented. Reported differences will include standard errors and 95% CI.

#### Data Storage

##### Data Management

Designated study site staff will directly query EPIC and retrieve a set of defined data fields that can be directly integrated into INOVA’s clinical or translational research database, REDCap. Specific forms will be used for each component of the subject’s progress. The forms and data dictionary will be available online for all individuals who perform data entry. Research personnel, trained on data definitions will perform logical data checks to assess data quality. Suspect data entries will be flagged for review and confirmed by the investigative team at each site.

Information about study subjects will be kept confidential and managed according to the requirements of the Health Insurance Portability and Accountability Act of 1996. Privacy and confidentiality of all patients enrolled will be maintained. Steps will be taken to “de-identify” participants from their personal health information (PHI) by assigning each patient a PIN number that will not be linked to any specific PHI. The “key” linking the PIN to the patient’s PHI will be maintained separately from the collected data and will be stored on a protected data file on a secure internet server requiring password login.

##### Records Retention

The investigator will maintain records in accordance with ICH Guidelines. Essential records will be stored for no longer than 3 years after the study is formally discontinued and then destroyed. Paper records will be shredded and recycled. Records stored on a computer hard drive will be erased using a commercial software application designed to remove all data from the storage device.

### Human Subjects Protection (Risks, Benefits, and Alternatives)

#### Risks

There are no anticipated significant risks in this study. Potential loss of confidentiality will be minimized by shielding the participants by unlinking his or her identity from his or her personal health information

#### Benefits

There are no direct benefits to the patient.

#### Confidentiality

The Principal Investigator and Coinvestigators will be ultimately responsible for assuring the security of all computer systems to minimize risk to participants. The participant’s identifiable private information will be handled, managed, and disseminated in a method which places confidentiality as the highest priority. Individuals who will have access to the data will need to be clearly delineated. The data will be stored in a Health Insurance Portability and Accountability Act of 1996 (HIPPA) compliant database, shared only with individuals who are participating in the study, and will be stored for no longer than 3 years and then eventually destroyed.

### Subject Compensation

#### Costs and Payment

There are no costs to participate in the study. There will be no payment for participation in this study.

### Adverse Event Reporting

There are no potential adverse events for participation in this study.

## Results

Preliminary data analysis for the year 2017 has been completed. Compared to a baseline 2016 survival rate of 47.0%, from 2017 to 2018, CS survival rates were increased to 57.9% (81/140) and 81.3% (26/32), respectively (*P*=.01 for both). Survival in acute MI with CS increased from 52.6% (30/57) to 75.0% (12/16) and in acute decompensated HF from 61.4% (51/83) to 87.5% (14/16). For 2017, CS threshold markers at 12 hours (lactate<3.0 mg/dL, CPO>.6 W, PAPi>1.0) overall survival was 80% (72/90), 93.6% (73/78) and 74% (71/96), respectively. For 2018, CS threshold markers at 12 hours (lactate<3.0 mg/dL, CPO>.6 W, PAPi>1.0), overall survival was 92.3% (24/36), 92% (23/25) and 85.2% (23/27), respectively. Use of RHC was associated with 14% greater survival. Decreases of 5 and 10 hours in the time to implement MCS were associated with increased survival 53.6% and 135.8%, respectively. Age was associated with survival; patient’s ≥75 years old had higher risk of death (odds ratio 3.43, 95% CI 1.20-9.78).

## Discussion

Retrospective clinical outcomes (acute decompensated heart failure cardiogenic shock, acute myocardial infarction cardiogenic shock) demonstrated an increase in current survival rates when compared to pre-Cardiogenic Shock team initiation. IHVI site specific positive outcomes far exceeded the national average. Patients who had mechanical circulatory support did significantly better when the patient was younger than 75 years old at the time of intervention and when the mechanical circulatory support was initiated within five hours of the patients’ arrival at the IHVI. Yet to be analyzed is the significance of the type of intervention ECMO versus intracorporeal axial-flow (Impella).

## Abbreviations

ACS: acute coronary syndrome
ADHF: acute decompensated heart failure
AMI: acute myocardial infarction
CS: cardiogenic shock
CPO: cardiac power output
ECMO: extracorporeal membrane oxygenation
HIPPA: Health Insurance Portability and Accountability Act
ICU: intensive care unit
IHVI: Inova Heart and Vascular Institute
MI: myocardial infarction
NSTEMI: non-ST-segment elevation myocardial infarction
PHI: personal health information
RHC: right heart catheterization
STEMI: ST-segment elevation myocardial infarction
UID: unique identification
